# A Case of Anti-Leucine-Rich Glioma-Inactivated Protein 1 (Anti-LGI1) Encephalitis With an Unusual Frontomesial Motor Cortex T2 MRI Hyperintensity

**DOI:** 10.7759/cureus.30480

**Published:** 2022-10-19

**Authors:** Giulio Papiri, Emanuele Puca, Matteo Marcucci, Cristina Paci, Claudia Cagnetti

**Affiliations:** 1 Neurology Unit, Ospedale Provinciale “Madonna del Soccorso”, San Benedetto del Tronto, ITA; 2 Department of Radiology, AOU Ospedali Riuniti "Umberto I - G.M. Lancisi-G. Salesi" - Università Politecnica delle Marche, Ancona, ITA

**Keywords:** encephalitis syndrome, autoimmune epilepsy, lgi1 antibody autoimmune encephalitis, lgi-1, autoimmune limbic encephalitis

## Abstract

Anti-leucine-rich glioma-inactivated protein 1 (anti-LGI1) encephalitis is a rare autoimmune disorder, classified within limbic encephalitides, and characterized by seizures and subacute cognitive-behavioral impairment, mainly affecting short-term memory and usually involving temporo-mesial lobe structures.

We present a case of anti-LGI1 encephalitis characterized by focal right lower limb motor seizures and pyramidal signs and responsive to high-dose methylprednisolone. The patient developed an atypical left frontal lobe parasagittal T2 hyperintense lesion on MRI within one month of hospital admission, which has not been described previously in this disease to the best of our knowledge.

## Introduction

Anti-leucine-rich glioma-inactivated protein 1 (anti-LGI1) syndrome is a specific subtype of autoimmune encephalitis characterized by positivity for anti-LGI1 antibodies, either in serum or in cerebrospinal fluid (CSF); such antibodies target LGI-1, a component of the voltage-gated potassium channel molecular complex, and can disrupt synaptic functioning and elicit neuronal hyperexcitability; furthermore, their titers have been linked to more aggressive disease courses [1,2].

Its clinical spectrum might encompass a variety of presentations, with different degrees of severity; the core symptoms comprise subacutely worsening cognitive and neuropsychiatric symptoms associated with seizures. The earliest manifestations might include short-term memory impairment or various kinds of seizures. Faciobrachial dystonic seizures (FBDS), consisting of brief jerk-like facial and upper limb involuntary movements, are thought to be the most characteristic presentation of this disease and are seen in about half of the cases [1,2]. Other epileptic manifestations, including focal, generalized seizures and convulsive status epilepticus, might precede the onset of FBDS [3]. Other features include psychiatric disturbances like hallucinations, delusions, and anxiety as well as sleep disorders, dysautonomia, and hyponatremia [4].

The etiology of the disease is not well understood, although malignant neoplasms have been reported to be associated with anti-LGI1 antibodies in 3-11% of cases [1,5]. Definitive diagnosis relies on autoantibody detection either in serum or in CSF, which might result within normal limits for protein content and cell count [5]. In previous studies, specific immunological features, such as antibody subtypes, or specific HLA haplotypes, such as DRB1*07:01, have been associated with this disease [1].

Neuroimaging and electroencephalography (EEG) play a role as ancillary methods, as they might highlight morphological or electrical changes suggesting the involvement of the limbic system. No specific MRI or metabolic marker has been established to date, although medial temporal lobe involvement, either as T2 hyperintensities in MRI or as hypometabolism in FDG-PET, has been described in up to 70% of cases [6,7]. Temporal lobe dysfunction might be related to the development of symptoms such as amnesia, confusion, disorientation, anxiety, and temporal lobe seizures. Recent studies have also highlighted that brain metabolic derangement might also involve areas that are not altered in MRI [8-10].

As for the therapeutic aspects, seizures are considered scarcely responsive to antiepileptic treatment, while global satisfactory clinical responses have been described after immunosuppressive treatment. As observed in other autoimmune diseases, administration of high doses of systemic corticosteroids or intravenous immunoglobulin therapy has been associated with a good therapeutic response, although incomplete recovery at 12 months and relapses have been frequently reported [5,11,12], especially concerning cognitive symptoms [13]. Repeated courses of treatment have also been reported to be beneficial in patients not responding at first or those who relapse; long-term immunomodulant therapies, like rituximab, mycophenolate mofetil, azathioprine, tacrolimus, or cyclosporin have also been tried in nonresponsive cases [11,12].

As for prognosis in general, almost all patients achieve significant amelioration of symptoms after immunotherapy, although only about 45% of them remain symptom-free [11]. Functional independence, defined as a modified Rankin Scale score of 0-2, is achieved in about 80% of patients [14]. Relapses are associated with a worse global prognosis and are thought to occur in about 16% of cases, while mortality is estimated to be about 5% [2].

We report a case of a patient with anti-LGI1 encephalitis with CSF antibody positivity who developed focal right lower limb motor seizures associated with progressive cognitive impairment and an atypical frontomesial lesion in MRI; such a feature, to our knowledge, has not been described yet in this disease.

## Case presentation

**Figure 1:**
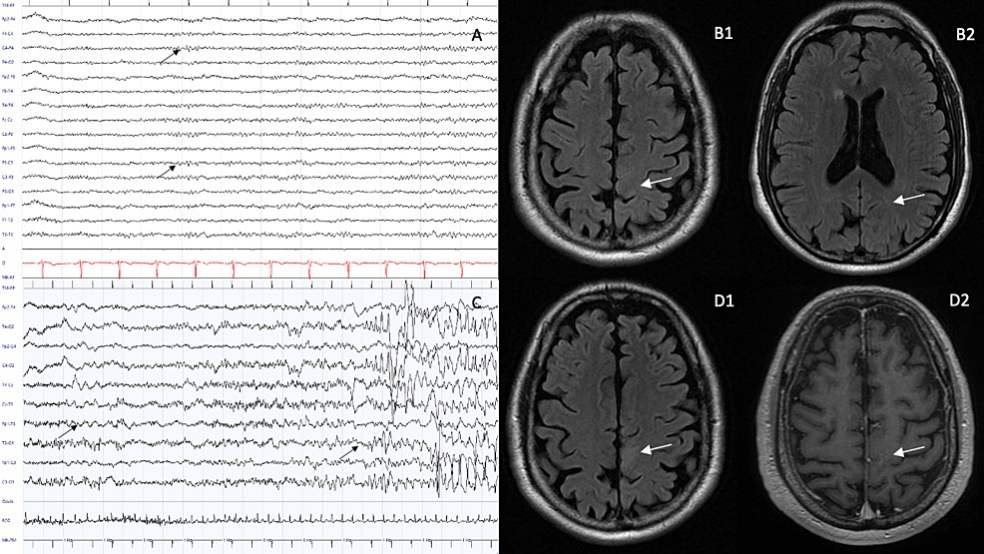
MRI and EEG findings EEG recording on hospital admission, showing only mild slowing in parasagittal chains (A); uncontrasted MRI FLAIR sequences, on day four after admission, showing normal findings in frontal mesial fields (B1, B2); EEG recording during a focal motor seizure with impaired awareness, showing diffuse slowing with left frontocentral slow and spiked activity with secondary generalization (C); MRI on day 11 after admission displaying a left frontomesial hyperintense lesion in T2 FLAIR sequences (D1) and mild contrast-enhancement (D2) MRI: magnetic resonance imaging; EEG: electroencephalography; FLAIR: fluid-attenuated inversion recovery

A 69-year-old male patient was referred to the emergency department after a generalized tonic-clonic seizure. His past clinical history was unremarkable. He denied any recent infections but reported sporadic occurrences of spontaneous brief muscle jerks in the right lower limb during the preceding week. He was afebrile and his blood chemistry workup was unremarkable. Emergency head CT was negative for acute hypo/hyperdense areas. He was referred to the local neurology ward; on admission, no focal signs were found and EEG displayed intermittent theta band activity in both parasagittal chains (Figure 1A); therefore, levetiracetam therapy (500 mg b.i.d.) was started, with benefit. On day four, 1.5 T uncontrasted brain MRI showed minimal nonspecific white matter hyperintensities, compatible with aspecific vascular leucoencephalopathy (Figure 1 - B1, B2).

On day five, the patient developed insomnia and night-time discomfort in the lower limbs, relieved by voluntary movement. Symptomatic treatment with low-dose clonazepam (0.5 mg OD) was added, with initial benefit. Over the following days, his short-term memory, orientation, and attention worsened. On day 10, involuntary right lower limb jerking movements appeared during the daytime. On examination, right lower limb (4/5) strength loss, urinary incontinence, ankle clonus, and homolateral extensor plantar response were observed. Continuous EEG monitoring revealed focal left frontocentral slowing and epileptiform discharges, synchronous with right lower limb jerking movements, with a tendency to contralateral spreading (Figure 1C). IV valproic acid (2000 mg per day) was added, with minimal benefit, although the overall condition did not deteriorate.

A subsequent contrast-enhanced MRI, on day 11, was negative for DWI-positive lesions, while showing a mild T2/FLAIR left frontal parasagittal hyperintensity (Figure 1 - D1). Based on a hypothesis of CNS inflammation, CSF testing was requested, which returned normal for chemical-physical properties and cell count. A CSF antibody panel was requested (anti-NMDA, anti-AMPA, anti-CASPR2, anti-Yo, anti-Hu, anti-Ri, anti-GABAB1, anti-DPPX, and anti-LGI1); a serum panel was not performed. A neck-chest-abdomen CT scan was negative for occult neoplasms. Neoplastic markers (alpha-fetoprotein, neuron-specific enolase, CYFRA 21-1, CA 125, CA 15-9, PSA, CA 72-4, and TPA) showed normal values. Screening tests for syphilis and Borrelia burgdorferi were negative. Antinuclear, anti-double-stranded DNA, anti-extractable nuclear antigen, anti-cyclic citrullinated peptide, and anti-phospholipid antibodies were not detected in serum. As per the hypothesis of an inflammatory disorder, a high-dose pulse steroid (methylprednisolone 1 g OD for five days) was initiated, yielding a moderate benefit, after a week, on mentation, gait, and a significant reduction in seizure frequency, accompanied by a complete recovery of right lower limb strength. The patient was discharged in a fully ambulatory condition, with mild memory and attention impairment, on day 21 since admission. His modified Rankin Scale score on discharge was 2.

A subsequent MRI, one month after hospital discharge, confirmed the presence of a left frontomesial contrast-enhancing lesion (Figure 1 - D2); immunological analyses subsequently revealed the presence of CSF anti-LGI1 antibodies, positive with a dilution titer of 1:32 (reference value: absent). The remaining antibodies in the panel were not detected. Eventually, the patient was lost to follow-up and we were not able to further assess his cognitive status.

## Discussion

The present case broadly fulfills the criteria for definite autoimmune encephalitis with anti-LGI1 antibody positivity, as per the guidelines laid out by Graus et al. [15]; lower limb focal motor seizures were the earliest and most prominent manifestation, followed by subacute neuropsychiatric impairment, characterized by prominent amnesia. In our case, symptoms were subtle and nonspecific at the beginning; only their deterioration over time, as well as the increase in seizure frequency, raised clinical suspicion of progressing disease. While FBDS is thought to be pathognomonic, other kinds of seizures as well as lower limb involvement have been documented in previous studies [5,16]. In neuroimages, temporo-mesial MRI T2 hyperintensity is considered common; however, as for other subtypes of autoimmune encephalitides, involvement of basal nuclei and insula has been reported, in both morphological and metabolic studies [6-9].

In our patient, MRI revealed an atypical frontomesial lesion in the left motor cortex, consistent with the epileptic focus, which might have been produced by inflammation and ictal discharges. In accordance with the hypothesis of immune-mediated epilepsy, antiepileptic drugs were ineffective in comparison to steroid therapy, which arrested seizure recurrence [5,7]. Furthermore, the left frontomesial lesion exhibited a variation in its appearance after steroid treatment, while no alteration in medial temporal fields was observed over time, despite the presence of amnesic symptoms.

The presence of high titers of anti-LGI1 antibodies in both CSF and serum has been linked to a more aggressive disease phenotype in comparison to seronegative cases, suggesting that a stronger immunologic response might be linked to a worse prognosis [1]. In accordance with this, status epilepticus at onset has been associated with a worse outcome, while the presence of MRI involvement has not been always associated with aggressive disease and its prognostic significance, as well as the presence of atypical lesions [2,3].

Prompt anti-inflammatory treatment has been associated with an overall better prognosis [6]; other prognostic markers are not well established, since data from longitudinal studies regarding the association between clinical phenotypes, MRI, metabolic, immunologic patterns, and prognosis in anti-LGI1 encephalitis are lacking [2,3]. Nonetheless, clinical phenotypes in this disease appear to be more variable than initially expected.

We recommend that further studies be conducted to elucidate the relationship between the involvement of specific brain areas, distinct clinical phenotypes, and prognosis in anti-LGI1 syndrome; as seen in our case, even if metabolic imaging was not carried out, the development of MRI changes a few days after the onset of seizures and cognitive dysfunction supports the hypothesis that focal or widespread immune-mediated alterations in brain energy metabolism might precede the appearance of inflammatory lesions and might therefore be the most pertinent manifestation of this disease.

## Conclusions

The present case, although its clinical course could be on the whole considered quite usual for autoimmune encephalitis, exemplifies the difficulties in diagnosing anti-LGI1 syndrome at the beginning of its course, especially if atypical radiological findings arise. Hence, clinicians should be aware of this disease when confronting late-onset epilepsy with predominantly focal motor seizures not responding to antiepileptic treatment, especially if accompanied by subtle cognitive deficits that quickly deteriorate. In fact, a high index of early suspicion remains crucial in order to achieve better clinical outcomes with anti-inflammatory treatment.
